# Activity capacity in children with early onset scoliosis compared to pulmonary function (spirometry) and patient-reported outcomes

**DOI:** 10.1007/s43390-025-01239-0

**Published:** 2025-12-09

**Authors:** Malvika Choudhari, Mark Belio, Di Hu, Stuart L. Mitchell, Joseph D. Stone, Erik D. Hanson, Feng-Chang Lin, Stephanie D. Davis, James O. Sanders

**Affiliations:** 1 School of Medicine, University of North Carolina at Chapel Hill, Chapel Hill, NC USA; 2 Department of Exercise and Sports Science, University of North Carolina at Chapel Hill, Chapel Hill, NC USA; 3 Department of Biostatistics, University of North Carolina at Chapel Hill, Chapel Hill, NC USA; 4 Department of Orthopaedics, University of North Carolina at Chapel Hill, Chapel Hill, NC USA; 5 Department of Pediatrics, University of North Carolina at Chapel Hill, Chapel Hill, NC USA

**Keywords:** Early onset scoliosis, Active gaming, Indirect calorimetry, Activity capacity, Metabolic equivalent, Pulmonary function

## Abstract

**Purpose:**

The primary concerning outcome in early onset scoliosis (EOS), pulmonary function, is challenging to measure in children. Surrogate measures, including thoracic length and curve magnitude, poorly predict patient outcomes. Activity capacity as determined by Metabolic Equivalents of Task (MET) is a potentially useful alternative or adjunct since it more directly reflects the tasks being performed. The objective of this pilot study was to assess MET values for varying intensity activities in children with EOS and explore their relationship to pulmonary function indices, scoliosis characteristics, and patient-reported outcome measures.

**Methods:**

For this pilot study, basal metabolic rate and physical activity MET values were measured using indirect calorimetry. MET values were assessed while performing video games representing low (bowling), moderate (boxing), and high intensity (active running) activities, and treadmill walking at low, moderate, and high intensity. MET values were compared to age-matched standardized energy expenditure values in youth (METy) for similar tasks. Pulmonary function testing was assessed using spirometry with percent predicted values based on arm span. Patient outcomes were obtained for each participant through the 24-question Early-Onset Scoliosis Questionnaire (EOSQ-24) and select assessments from the Patient-Reported Outcomes Measurement Information System (PROMIS). A linear mixed model assessed differences between groups. Spearman correlation coefficients assessed relationships between variables.

**Results:**

Eight children (ages 6-16y, 50% female) completed testing. Etiologies for scoliosis were congenital (*n* = 3), syndromic (*n* = 3), and idiopathic (*n* = 2). Five had spirometry suggestive of severe restriction (FVC and FEV_1_ < 50% predicted). Children with EOS had a 0.6 lower mean MET video game value compared to published METy values (*p* < 0.001). Increased intensity corresponded with increased MET values comparing hard to moderate and low intensities (*P* < 0.001). Average percent predicted MET values across all tasks revealed a negative correlation with FEV_1_/FVC (*R* = − 0.927, *p* = 0.024). All eight children completed the low-intensity treadmill and low and moderate-intensity videogames; seven completed the moderate-intensity treadmill and high intensity videogame. Only five completed the high-intensity treadmill.

**Conclusion:**

MET values in children with EOS were directionally similar to values in normal children. Children unable to generate higher MET values appear to self-limit their activity. The relationship between MET and pulmonary function is complex and requires further exploration. Children with EOS and pulmonary impairment appear unable to generate the energy required for more vigorous activities. This could be due to poor pulmonary function, deconditioning from their underlying condition, or limitations in the tested video games.

**Level of evidence:**

II.

## Introduction

Morbidity and mortality from early onset scoliosis (EOS) are usually from pulmonary impairment. However, evaluating pulmonary impairment may be challenging; children with impaired cognition may have difficulty performing pulmonary function testing (PFT) and children < 6 years cannot typically reliably perform spirometry. Surrogate measures, including thoracic length and curve magnitude, are poorly predictive of patient outcomes [1]. Parameters accurately reflecting lung function or respiratory status are needed to monitor the disease course, thereby leading to interventions that improve outcomes.

Metabolic equivalents of task (MET) is a potential alternative or adjunct to PFT. MET is the energy consumption during an activity compared to resting energy consumption or Resting Metabolic Rate (RMR). Higher MET indicates higher activity energy requirements. Published for a wide range of activities in healthy youth (METy) [2], METs are comparable between activities, and METs or the associated activities may be easier to measure and track in children than PFTs. As a true energy consumption measure, METs may provide more useful functional measurements of EOS improvement or deterioration than other measures. However, it is unclear whether METs are valid in EOS, associated with PFT values, or reliably measure children’s capabilities. Given the rarity and heterogeneity of EOS, establishing MET feasibility across the spectrum is a critical step in evaluating its potential as a functional capacity measure in EOS.

The primary objective of this pilot study is to explore whether MET values in children with EOS are equivalent and ordinally similar to published METy values, that is, activities requiring more energy in healthy children likewise require more energy in children with EOS. The secondary objective is to explore the association of PFT values, scoliosis characteristics, patient-reported outcome measures (PROM), and MET values. We hypothesized that pulmonary impairment limits physical activity, and that this limitation is either reflected in increased task-specific MET values compared to healthy children or an inability to expend the energy necessary for a task.

## Materials and methods

Informed consent and/or assent were obtained for this institutional review board-approved study. Inclusion criteria were: (1) ages 6–18 years, (2) any curve pattern impacting the thoracic or lumbar spine, (3) spinal radiographic imaging within 6 months of testing, and (4) parent able to communicate in English or via translator. Exclusion criteria were: (1) known uncorrected cardiac disorder, e.g., cardiac myopathy or congenital cardiac anomaly likely to affect cardiac output capacity, (2) treatment with bilevel positive airway pressure, (3) inability to understand instructions for exercise or spirometry (e.g. severe autism/cognitive problems) and (4) any other abnormalities potentially impacting pulmonary function or MET testing, such as metabolic disorders or limb dysfunction.

Participants completed the Patient-Reported Outcomes Measurement Information System (PROMIS) Pediatric Profile-25 [3], Pediatric Sleep Disturbance [3, 4], and Pediatric Sleep-Related Impairment questionnaires, while parents completed the 24-question Early-Onset Scoliosis Questionnaire (EOSQ-24) [5] and proxy versions of the sleep assessments.

PFTs were performed by a pediatric pulmonary function technician using American Thoracic Society(ATS) and European Respiratory Society(ERS) standards [6] and interpreted by a pediatric pulmonologist. Spirometry was obtained using a ComPas™ portable system *(Morgan Scientific, Haverhill, MA)* [6]. The parameters assessed were baseline respiratory rate, 1 s forced expiratory volume (FEV_1_), forced vital capacity (FVC), forced expiratory flows between 25 and 75% of FVC (FEF_25-75_), and FEV_1_/FVC ratio. Each test was performed 3–8 times. All lung function parameters were analyzed and accepted per ATS and ERS standards [6]. Percent predicted lung function values were based on international normative data derived from arm span [7, 8]. 

MET testing was conducted by an exercise physiologist. RMR was assessed as volume of oxygen consumed (VO_2_) in milliliters of oxygen per kilogram of body mass per minute (mL/kg/min) from respiratory gases analyzed using a metabolic cart (*TrueMax 2400, Parvo Medics, Salt Lake City, UT*). Following flowmeter and gas calibrations, each participant was fitted with a properly sealed face mask (*Hans Rudolf, Kansas City, MO*). Participants rested supine breathing normally for 5 min. RMR was determined as the average VO_2_ during the last rest minute.

Following RMR testing, participants were familiarized with the Nintendo Switch™ video game system. All activities were first demonstrated. Participants practiced on the low intensity setting until comfortable playing. The mask was re-applied, and following a standardized rest period, the participant performed the active video game activity for 3 min while respiratory gases were collected. MET for each activity was calculated as:

MET = VO_2_ of activity (mL/kg/min) averaged during the last 30 s of each task.

Nintendo Switch active video games were selected to correspond with published METy activities of low (< 3 MET), moderate (3–6 MET), and hard (> 6 MET) intensity. For the hard intensity, Action Running was selected as it had the highest listed METy value. Table 1 shows the selected video games, corresponding intensity, METy category, and values for each age-group category, including various treadmill walking speeds [2]. EOS-assessed MET values were compared to age-matched compendium METy values for similar tasks.

**Table 1:** Youth compendium metabolic equivalents of task grouped by activity and age group

Compendium code	Activity	METy 6–9y	METy 10–12y	METy 13–15y	Intensity	Nintendo switch game
20100X	Active Video Games—Bowling	2.1	2.3	2.4	Low	Nintendo Switch Sports—Bowling
15140X	Active Video Games—Boxing	3.0	4.0	4.9	Moderate	Fitness Boxing 2
15100X	Active Video Games—Action Running	4.8	5.9	6.8	High	Active life outdoor challenge
80120X	Walk 1.0 mph	2.5	2.6	2.7		
80140X	Walk 1.5 mph	2.5	2.7	2.9		
80160X	Walk 2.0 mph	2.8	3.0	3.2		
80180X	Walk 2.5 mph	3.3	3.5	3.6		
80200X	Walk 3.0 mph	3.8	4.1	4.3		
80220X	Walk 3.5 mph	4.6	5.0	5.3		
80240X	Walk 4.0 mph	4.8	5.2	5.6		

Adapted from Butte 2018. *METy* Metabolic equivalents of task in the youth compendium for healthy children

The Bowling game was performed in single-player mode with participants swinging their arm as if rolling a bowling ball. Fitness Boxing 2 was played at the simplest difficulty. Active Life Outdoor Challenge is a running and jumping game where the participant played the ‘5-Minute Hard Exercise Training’ mode for 3 min. Participants had a controller strapped to their left leg and either stepped in place or lifted their left knee up to have their character run forward on the track or jump over objects in their path, respectively. Participants were instructed to step at a comfortable pace they could maintain for 3 min. The activity was discontinued if they could not complete it or was deemed above their capabilities.

Participants then practiced walking on a treadmill (*T2100, General Electric Medical Systems Information Technologies INC., Milwaukee, WI*) until comfortable. Participants walked at various speeds for 30 s each and provided their rate of perceived exertion (RPE) using the EP7 scale [9, 10]. Based on the provided practice RPE, perceived participant capabilities, and METy values similar to the corresponding active video game intensity, the treadmill speed was individually determined for low, moderate, and hard intensities in order following a standardized 5 min rest period. For each intensity, participants walked at the selected speed with a 0% grade for 3 min while wearing the face mask. The last 30 s of respiratory gas data were averaged to compute the activity MET.

### Statistical analyses

Descriptive statistics were computed for all variables, including means and standard deviations for continuous variables and frequencies and percentages for categorical variables. A linear mixed model was used to assess differences in MET between groups, with activity and intensity as fixed effects and participants as random effects to account for dependency in repeated measurements. The model was estimated using restricted maximum likelihood (REML), and hypothesis testing for differences between intensity groups was conducted using F-tests. The Bonferroni method was applied to control the family-wise type I error. Degrees of freedom for the approximate F-tests were estimated using the Kenward-Roger method. Spearman correlation coefficients assessed relationships between variables. For correlations, EOS-derived and direct MET values were also transformed into percent predicted METy values to account for variation in age. Paired t-tests were conducted to compare EOS-derived MET values with age-matched normative METy values for each activity and intensity level. All tests were two-tailed, with statistical significance set at *p* < 0.05. Analyses were performed using Jamovi version 2.3.28 (Sydney, Australia), an open-source statistical software package, and SAS 9.4 (Cary, NC).

This manuscript is reported following STROBE (Strengthening the Reporting of Observational Studies in Epidemiology) guidelines [11].

## Results

**Fig. 1:**
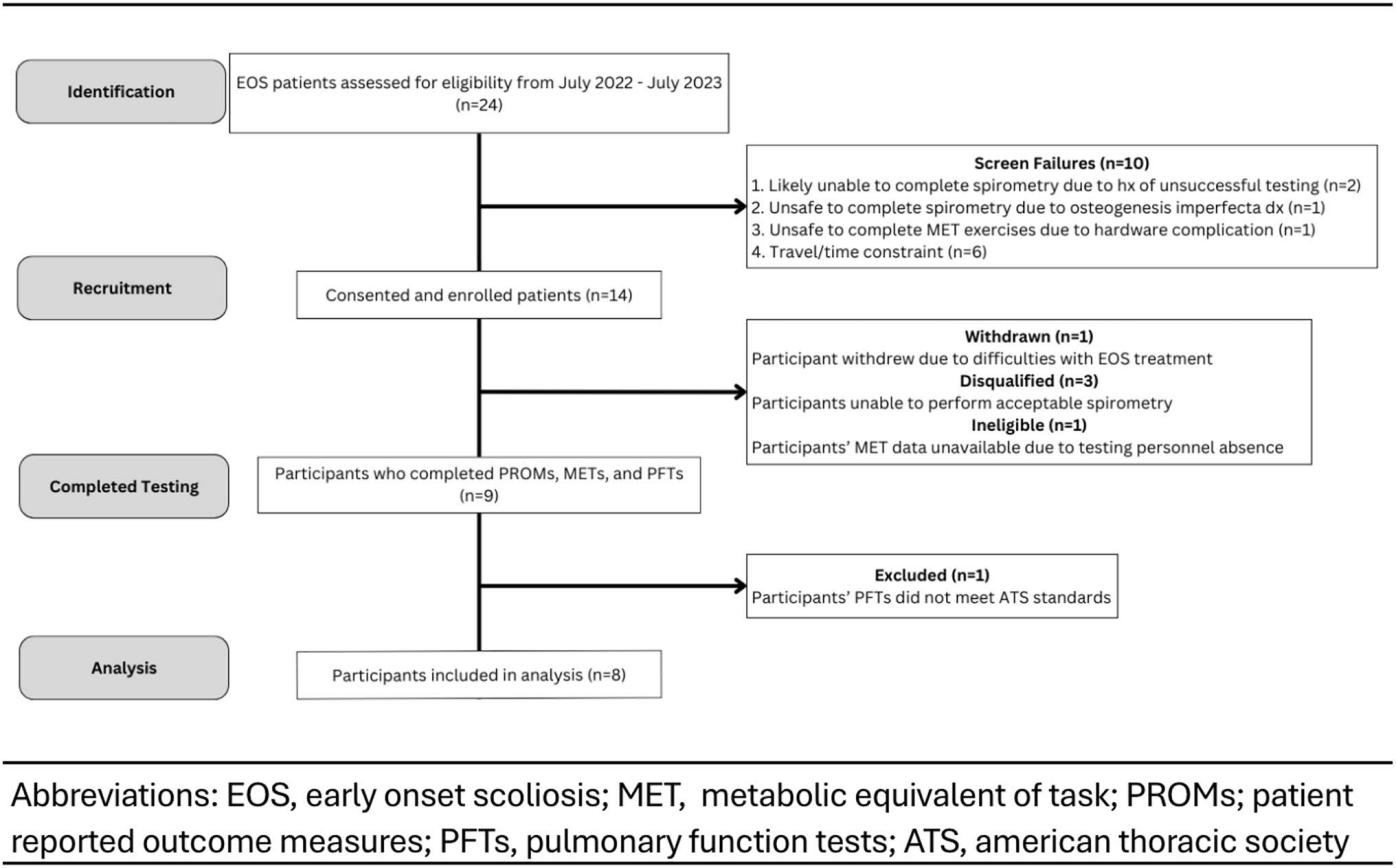
EOS Participant Flow Diagram from Identification to Analysis. *EOS* early onset scoliosis, *MET* metabolic equivalent of task, *PROMs* patient reported outcome measures, *PFTs* pulmona function tests, *ATS* American thoracic society

24 children with EOS were identified for this study and eight participants (ages 6–15 years, 50% female) completed testing (Fig. 1). Participant characteristics are described in Table 2. Participants had prior surgery (3), bracing (3), casting (1) or observation (1). Etiology was congenital (3), syndromic (2), idiopathic (2), and neuromuscular (1). The average major curve magnitude for participants was 63° at the time of study (range 35°–138°), with five participants < 50°, two with prior surgery, and three > 50°, one with prior surgery.

**Table 2:** Subject characteristics (*n* = 8)

Age (years)	Sex	Height (cm), percentile	Weight (kg), percentile	Diagnosed comorbidities	Etiology	Prior treatment	Major cobb angle (°)	T1-12 (cm)	FVC % Predicted	FEV1%. predicted	FEF_25-75_% predicted	FEV1/FVC ratio
6	F	116.5, 29	21.3, 43	Syringomyelia	Congenital	None	28 MT, 38 TL	15.6	88	90	96	92
6	M	128, 80	24, 68	OSA	IIS	Casting	47 MT	18.9	102	105	116	89
7	F	106, 0.1	15.8, 0.2	Tethered Cord	Congenital	Brace	88 MT	15.5	45	40	20	81
9	M	128.4, 9.3	21.2, 0.4	Congenital Myopathy, OSA	Neuromuscular (NAM)	Halo Tx, Growing rods	35 MT	17.1	45	38	26	74
11	F	145, 58	29.9, 10	None	IIS	Brace	44 MT	19	93	79	46	75
11	M	133, 1.5	25.4, 0.3	Pectus Carinatum, OSA, mild asthma	Syndromic (NF1)	Halo Tx, Vasc Rib, Shilla	54 UT	17.4	42	43	49	88
12	F	151, 48	34.2, 14.5	Pectus Carinatum, Restrictive lung Disease	Syndromic (Beals)	Casting, Growing rods, PSIF	48 MT	20.7	39	41	66	93
15	M	148, 0.3	63.8, 71	Asthma	Congenital	None	86 UT, 101MT	15.8	35	32	25	80

Percent predicted lung function values were based on international normative data derived from arm span

*IIS* infantile idiopathic scoliosis, *NAM* native american myopathy, *OSA* obstructive sleep apnea, *PSIF* posterior spinal instrumentation and fusion, *MT* Main Thoracic, *UT* Upper Thoracic, *TL* Thoracolumbar, *FVC* forced vital capacity, *FEF* forced expiratory flows, *FEV1* forced expiratory volume in 1 s

Results of pulmonary function testing demonstrated five of the eight participants with spirometry suggestive of severe restriction with both FVC and FEV_1_ less than 50% predicted (Table 2**)**.

MET testing results are presented in Table 3**.** All participants were able to complete the low-intensity activities (bowling and walking) and the moderate-intensity gaming (boxing) activity. Seven completed the high-intensity gaming activity (action running) and moderate treadmill walking. Five completed the hard treadmill activity. Using the overall average values for video games and treadmill, children with EOS had a 20% lower MET value compared to published METy values (mean difference − 0.6, *p* < 0.001) with this difference primarily driven by video games, where MET values were 48% lower (− 1.2, *p* < 0.001) with no difference present during walking tasks (− 0.1, NS). When examining individual tasks, both the low (− 0.7, *p* < 0.001) and moderate (− 1.8, *p* < 0.001) intensity gaming activities were significantly lower than corresponding published METy values, while there was no difference for the other four tasks. The results of the linear mixed model indicate that an increase in intensity was associated with a higher MET value when comparing hard intensity to moderate and low intensities (*P* < 0.001). However, no significant difference was observed between moderate and low intensities (*P* = 0.319). A similar effect of intensity was observed for treadmill walking. Detailed results from the linear mixed model are presented in Table 4.

**Table 3:** Comparison of metabolic equivalents of task across various active gaming and treadmill activities and intensities in children with early onset scoliosis vs published values in youth compendium

Mode	Intensity	Specific activity	Participants performed (% of total)	EOS MET values (Mean ± SD)	Healthy children METy values (Mean ± SD)	Mean difference (95% CI)
Active gaming	Easy	Bowling	8 (100%)	1.5 ± 0.2	2.2 ± 0.1	− 0.7 (− 0.9, − 0.5)*
Active gaming	Moderate	Boxing	8 (100%)	1.9 ± 0.7	3.7 ± 1.0	− 1.8 (− 2.5, − 1.2)*
Active gaming	Hard	Action running	7 (88%)	4.5 ± 1.6^^#^	5.3 ± 0.6	− 0.8 (− 2.0, 0.5)
			Active gaming average	2.5 ± 1.6	3.7 ± 1.4	− 1.2 (− 1.5, − 0.7)*
Treadmill walking	Easy	Walking	8 (100%)	2.3 ± 0.7	2.6 ± 0.1	− 0.3 (− 0.8, 0.3)
Treadmill walking	Moderate	Walking	7 (88%)	3.0 ± 0.8	3.3 ± 0.2	− 0.3 (− 1.2, 0.5)
Treadmill walking	Hard	Walking	5 (62%)	5.0 ± 1.4^^#^	4.6 ± 0.4	0.4 (− 1.5, 2.1)
			Treadmill average	3.2 ± 1.4	3.3 ± 0.9	− 0.1 (− 0.5, 0.3)
			Overall average	2.9 ± 1.6	3.5 ± 1.2	− 0.6 (− 0.3, − 0.9)*

*EOS* early onset scoliosis, *MET* metabolic equivalent of task, *METy* metabolic equivalent of task in youth compendium

^*^p < 0.05 EOS MET vs healthy children METy

^*p* < 0.05 easy vs hard intensity

^#^*p* < 0.05 moderate vs hard intensity

**Table 4:** Detailed analysis results from the linear mixed model

Effect	Estimate (SE)	95% CI	*P* value	Adjusted *P* value
Intercept	2.3 (0.3)	(1.75, 2.88)	< .0001	< .0001
Activity—treadmill walking vs active gaming	− 0.8(0.3)	(− 1.42, − 0.24)	0.0073	0.0073
Intensity—hard vs easy	2.8(0.4)	(2.07, 3.55)	< .0001	< .0001
Intensity—moderate vs easy	0.5(0.3)	(− 0.20, 1.18)	0.1595	0.3191
Intensity—moderate vs hard	2.3(0.4)	(1.57, 3. 06)	< .0001	< .0001

**Fig. 2:**
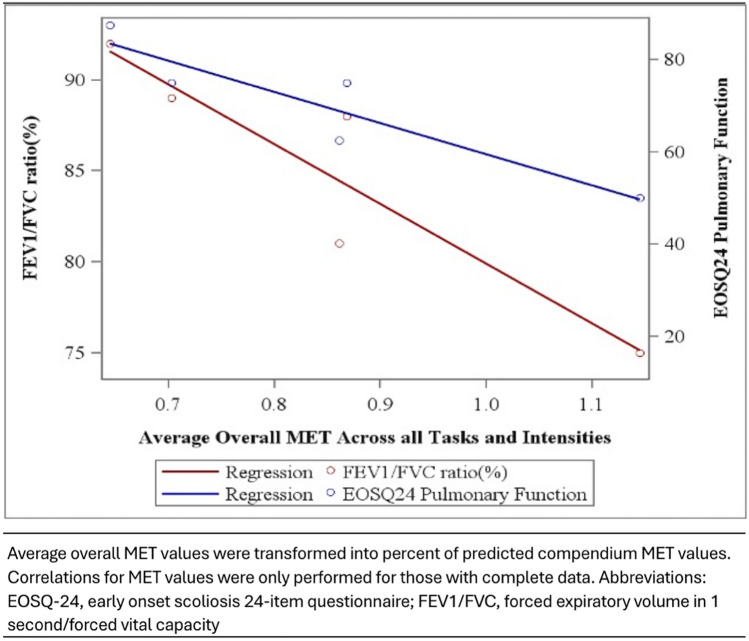
Correlations between Metabolic Equivalents of Task, Pulmonary Function Testing, and Patient Reported Outcomes in Child ren with Early Onset Scoliosis. Average overall MET values were transformed into percent of predicted compendium MET values. Correlations for MET values were only performed for those with complete data. *EOSQ-24* early onset scoliosis 24-item questionnaire, *FEV1/FVC* forced expiratory volume in 1 second/forced vital capacity

Absolute MET values did not correlate with PFT values. Correlations between average METs and FEV_1_/FVC are presented in Fig. 2. However, average percent predicted MET values (MET:METy equivalent) across all tasks had a strong negative correlation with FEV_1_/FVC (*R* = − 0.927, *p* = 0.024); particularly for treadmill tasks. There were no other significant correlations between outcomes.

EOSQ24 average scores were: 70 (range, 41–91) overall, 63 (range, 13–100) for pulmonary function, 81 (range, 42–100) for physical function, and 61 (range, 25–100) for fatigue/energy. PROMIS Profile-25 average scores in the EOS population were similar to the calibration population scores, but with individual variability. Sleep scores followed this trend, except for participants’ sleep disturbance (mean 53.4; range, 36.6–61.4) and parent proxy sleep impairment (mean 52.6; range, 43.8–60.2) both meeting the minimally important difference (MID ≥ 3 points). Significant correlations (*p* < 0.05) were observed between proxy-reported sleep disturbance and MET values across all tasks and intensities (*n* = 5, *r* = 0.985), as well as for treadmill-based MET values (*n* = 5, *r* = 0.967). Additionally, a strong correlation was found between proxy-reported sleep-related impairment and MET values across all video game activities (*n* = 7, *r* = 0.859). A notable negative correlation was observed between the EOSQ Pulmonary domain score and MET values during video game activities (*n* = 7, *r* = − 0.782), suggesting that worse pulmonary function was associated with lower MET values (Table 5).

**Table 5:** Spearman correlations between metabolic equivalents of task, maximum treadmill speed, and patient reported outcomes in children with early onset scoliosis

Patient reported outcome measure	EOSQ-24	PROMIS Pediatric Profile-25 Domains						PROMIS sleep assessment			
	Pulmonary function domain	Physical function mobility	Anxiety	Depression	Fatigue	Peer relationships	Pain interference	Peds sleep disturbance	Peds sleep-Related impairment	Parent proxy sleep disturbance	Parent proxy sleep-related impairment
Average MET Across all Nintendo Switch Activities (*n* = 7)	− 0.7	0.1	0.4	0.0	− 0.2	0.4	− 0.3	0.6	− 0.2	0.6	0.757*
Average MET Across all Treadmill Activities (*n* = 5)	− 0.8	− 0.5	0.1	− 0.6	− 0.1	0.5	− 0.4	0.3	− 0.9	0.949*	0.6
Average Overall MET Across all Tasks and Intensities (*n* = 5)	− 0.8	− 0.5	0.1	− 0.6	− 0.1	0.5	− 0.4	0.3	− 0.9	0.949*	0.6
Maximum Treadmill Speed Tested that Participant Could Complete (*n* = 8)	0.3	0.3	− 0.5	− 0.2	0.4	− 0.4	− 0.6	− 0.2	− 0.6	0.2	0.3

Average MET values were transformed into percent of predicted compendium MET values. Correlations for MET values were only performed for those with complete data for the relevant tasks

*EOSQ-24* early onset scoliosis 24-item questionnaire, *PROMIS* Patient-Reported Outcomes Measurement Information System, *MET* metabolic equivalent of task

^*^p < 0.05

## Discussion

Children with EOS vary widely in etiology, pulmonary impairment, musculoskeletal comorbidities, and developmental capability, all of which influence functional performance. Because of thoracic shape distortion, EOS can create respiratory compromise. Unfortunately, with no direct relationship between scoliosis parameters and measured pulmonary function and challenges obtaining PFTs in young children, who often have the worst disease, standard measures of scoliosis and pulmonary function are problematic for the most important parameter, namely how EOS effects adequate respiratory exchange. Indeed, six potential participants in this study were either ineligible or disqualified because of their inability to perform acceptable spirometry.

Surrogates for pulmonary function testing include thoracic height [12], 6-min walk test [13, 14], CT lung volumes [15], dynamic MRI [16–20], and motion analysis [21–24]. While each of these captures a different domain of overall pulmonary status, functional tests such as the 6-min walk test, one of several “field walking tests”, are those most directly tied to physical function. The standard 6-min walk records distance walked, number of stops, total time stopped, SpO2 nadir, and end-test pulse rate [25–27]. Oxygen desaturation and systolic blood pressure increases do correlate with curve severity [28].

MET, the ratio of the energy used, specifically O_2_ consumption, when performing a task compared to rest, has not been evaluated in EOS. Any task, such as the distance walked in 6 min, requires effort which can be measured in METs, but MET is a more generalizable measure and provides a standard that can be applied across activities and etiologies. Inability to deliver oxygen effectively because of respiratory insufficiency will limit the ability to generate MET required for high-energy activities. Playing a low-demand video game requires a specific MET and running a hard sprint a different, higher MET. MET, being measurable energy consumption, potentially allows comparisons between tasks in children who cannot perform specific tasks by substituting another activity. For example, a child who cannot walk can potentially perform another task with their arms and still have a comparable measure.

Because this is a relatively small pilot study, our focus was demonstrating the feasibility of measuring METs in this population and identifying important correlations warranting deeper exploration. Our subjects were mostly children with severe restrictive lung disease, with five of the eight having FEV_1_ and FVC < 50% predicted. In this cohort, we did not find a direct correlation between PFTs and MET production, though when we compared FEV_1_/FVC to the percent predicted MET (MET/METy), we found a strong association between worsening FEV_1_/FVC and MET indicating that in children with evidence of mixed obstructive and restrictive lung disease, MET-generating ability was worsened.

Unlike the first part of our hypothesis that MET would increase in activities in children with EOS, we found that children limit their activities rather than generate higher MET which was the second part of our initial hypothesis. While all participants completed the low treadmill, easy gaming (bowling), and medium gaming (boxing) activities, only seven completed hard gaming (running) and moderate treadmill activities, and five the hard treadmill activity. With the video games in the study, participants could decrease their effort and still complete the task (boxing), a limitation of the selected games. It is likely other games can be developed better separating children based on their capacity. Additionally, it is important to note that the overall MET to METy difference of 0.6 was driven by the video game task, as treadmill values were quite similar to METy values. Overall, measured METs were directionally similar to corresponding METy.

While PROMs purportedly assess children's functional capacity, only one, the EOSQ24, specifically attempts to assess EOS pulmonary and energy deficiencies [29, 30]. Its two questions on pulmonary function ask, “How difficult has it been for your child to cry/gurgle/talk without having shortness of breath?” and “How often has your child been having shortness of breath during physical activity?”. The energy questions ask, “How often was your child exhausted?” and “How difficult was it for your child to keep his/her energy level throughout the day?” Neither of these reflects children self-limiting activity to avoid shortness of breath or exhaustion, which may explain the lack of significant correlation between MET and the EOSQ24 pulmonary function domain. The selected PROMIS measures [31–33] indicate that, on average, the participants’ self-reported and proxy-reported level of function were at a similar level to the calibration populations, with some variability, and certain measures meeting the MID of ≥ 3 points. When assessing the correlation of the PROMIS Profile25 and sleep measures, we did not find any association with MET or maximum treadmill speed. This finding may be due to our small sample size in this pilot study or the reliance of these instruments on child-reported perceptions, which may not be perceived as true function limitations whereas METs quantify objective physiologic output. The PROMIS measures and EOSQ24 are self or parent/proxy reports rather than measurements of actual activities. Unfortunately, the self-reports may also not be inter-subject comparable, nor comparable across children with different underlying disorders [34]. Self-reported and proxy-reported activities differ from objectively measured activities, such as those assessed by MET. That said, we did find a high correlation between certain proxy sleep measures and MET suggesting that physiologic capacity may relate more closely to aspects of fatigue and sleep disturbance than to subject reports of daily function.

Findings from this study imply that children with impaired lung capacity from EOS demonstrate worse activity performance and have impaired pulmonary function compared to healthy children and, therefore, subsequently decrease their activities. This may be secondary to their lung capacity, increased work of breathing, weak and inefficient muscles [35, 36], or deconditioning from sedentary lifestyles [37–39]. It also showed that published METy values reflect those in children with EOS potentially making them helpful for future PROM development. From this pilot study, we suggest future work using MET in EOS should extend to children with less severe scoliosis, including normal controls, further quantifying relationships between pulmonary function, thoracic distortion, and activity capacity (particularly how children self-limit their activity and how or whether intervention improves this capacity), additional sleep measures and improved gamification of MET. A key finding from this study is children's activity self-limitation. If this holds with a larger study, it suggests the potential value of METs in understanding the actual capabilities and behaviors of children with significant EOS.

## Data Availability

Data used in this study will be provided upon request.
